# The Various Substances Resembling Membranes Found in the Throats of Children

**Published:** 1887-03

**Authors:** 


The Various Substances Resembling Membranes found in the Throats of Children.—According to the famous pædiatrician, J. Simon, these products are as follows:

1. Diphtheritic products embedded in the mucous membrane and firmly attached to it by fibrous prolongations. The fibrous nature of this membrane may be demonstrated microscopically. These exudations are usually accompanied by enlargement of the submaxillary glands and slight rise of temperature.

2. Pultaceous products, that is, epithelial substances with mucous.

3. Herpetic products, often found in connection with tonsillitis. These are all soluble in water.

4. Products resulting from cauterization.

5. Milk deposits composed principally of casein and often found in the throats of nursing infants.

6. *Muguet* or thrush, and the diphtheroid angina of children having exhausting diseases, such as typhoid fever, severe bronchitis, scarlatina. These deposits are often thrown out within twenty-four hours, as are those of true diphtheria. The subsequent course of these diseases alone enable us to make an accurate differential diagnosis. When called to see a case in which the diagnosis of diphtheria is possible, I say that I consider the case non-malignant, but as we cannot be certain of this, the child should be treated as though he had a true diphtheria.—*El Siglo Medico.*

